# Variations of *CITED2* Are Associated with Congenital Heart Disease (CHD) in Chinese Population

**DOI:** 10.1371/journal.pone.0098157

**Published:** 2014-05-21

**Authors:** Yan Liu, Fengyu Wang, Yuan Wu, Sainan Tan, Qiaolian Wen, Jing Wang, Xiaomei Zhu, Xi Wang, Congmin Li, Xu Ma, Hong Pan

**Affiliations:** 1 Graduate School, Peking Union Medical College, Beijing, China; 2 National Research Institute for Family Planning, Beijing, China; 3 Henan Research Institute of Population and Family Planning, Key Laboratory of Population Defects Intervention Technology of Henan Province, Zhengzhou, China; 4 Cardiac Surgery Department, Xiamen Heart Center, Organ Transplantation Institute of Xiamen University, Xiang'an District, Xiamen, China; 5 Key Laboratory of Genetics and Birth Health of Hunan Province, Family Planning Institute of Hunan Province, Chang sha, China; 6 Department of Medical Genetics, School of Basic Medical Sciences, Capital Medical University, Beijing, China; 7 World Health Organization Collaborating Centre for Research in Human Reproduction, Beijing, China; Northwestern University, United States of America

## Abstract

*CITED2* was identified as a cardiac transcription factor which is essential to the heart development. *Cited2*-deficient mice showed cardiac malformations, adrenal agenesis and neural crest defects. To explore the potential impact of mutations in *CITED2* on congenital heart disease (CHD) in humans, we screened the coding region of *CITED2* in a total of 700 Chinese people with congenital heart disease and 250 healthy individuals as controls. We found five potential disease-causing mutations, p.P140S, p.S183L, p.S196G, p.Ser161delAGC and p. Ser192_Gly193delAGCGGC. Two mammalian two-hybrid assays showed that the last four mutations significantly affected the interaction between *p300CH1* and *CITED2* or *HIF1A*. Further studies showed that four *CITED2* mutations recovered the promoter activity of *VEGF* by decreasing its competitiveness with *HIF1A* for binding to *p300CH1* and three mutations decreased the consociation of *TFAP2C* and *CITED2* in the transactivation of *PITX2C*. Both *VEGF* and *PITX2C* play very important roles in cardiac development. In conclusion, we demonstrated that *CITED2* has a potential causative impact on congenital heart disease.

## Introduction

Congenital heart disease (CHD) is a most common defect caused by abnormal cardiac formation in fetuses and has become the leading reason of childhood mortality with an incidence around 1%[1]–[3]. In the past decades, a series of CHD-causing genes have been identified such as *NKX2-5*, *TBX5*, *GATA4* and *CITED2* [4]–[6]. It has been confirmed that their mutations can cause cardiac malformations through affecting the transcription activity of critical genes involved in heart development pathways.

*CITED2* (Cbp/p300-interacting transactivator, with Glu/Asp-rich carboxy-terminal domain, 2) is one member of a new conserved family of transcriptional activators which includes four members: *CITED1* (*Msg1*), *CITED2* (*Mrg1/p35srj*), *CITED3* and *CITED4* (*Mrg2*) [7]. *CITED2* is a nuclear protein which binds closely to the CH1 region of *p300* and *CBP* by its CR2 region (including a conserved 32-amino acid sequence [8]). Meanwhile, many other transcription factors and transcription regulating factors such as *HIF1A*, *RXRα*, *NFk*, *Mdm2*, *Ets-1* and *Stat2* also bind to the CH1 region of *CBP/p300* [9], [10]. Thus *CITED2* may act as a pivotal transcriptional modulator to regulate the expression of some specific genes. For example, *CITED2* decreased the expression of *HIF1A* (Hypoxia Inducible Factor 1) through its competitive binding to *CBP*/*p300CH1* [11], [12], consequently interfering the transcription of genes induced by *HIF1A* such as *VEGF* (vascular endothelial growth factor) [13]. It has been confirmed that the overexpression of *vegf* is the main factor resulting in cardiac malformation in *cited2* ^-/-^ mice [14].

Besides being a transcriptional repressor of *HIF1A*, *CITED2* acts as a transcriptional coactivator of *TFAP2* (transcription factor AP2, also called *Tcfap2*) [15]. Mutations of *TFAP2A* and *TFAP2B* result in neural tube, cranial ganglia defects and cardiac malformations [16], [17]. This suggested that the coactivation of *TFAP2* with *p300*, *CITED2* and *CREBBP* is essential for the normal development of those structures. As a critical transcription factor, *TFAP2* can affect the transcription of many genes, including *PITX2C* (Paired-Like Homeodomain 2 C)which is critical in Nodal-*PITX2C* pathways [18]. In addition, it has been detected that *TFAP2* isoforms and *CITED2* work together on the *PITX2C* promoter1 which controls the expression of *PITX2C* in the heart of embryonic mice. The mice experiments already indicated that knocking out *pitx2c* gene can lead to valve defects, body wall dysraphism, gastroschisis, ectopia cordis and other multiple organs polymorphous defects [19].

*CITED2* gene mutation in human congenital heart disease was first reported by Sperling *et al* [20] in 2005. They identified 3 mutations which alter the amino acid sequence and studied their association with *HIF1A* and *TFAP2C*. Their study confirms that *CITED2* is an important transcription factor in heart development and provides new insights into the molecular mechanism of congenital heart defects. Later, Yang *et al* found 3 new mutations in Chinese patients with congenital heart disease (2010) [21]and Chen *et al* [22]demonstrated another 3 new mutations in European CHD patients. Recently, Xu *et al* found 3 *CITED2* gene mutations, their research showed that *CITED2* gene mutations and methylation may play an important role in CHD. In their study, most of these mutations were in SRJ region. The mutations in our study were identified for the first time and located in SRJ region as well. Our work aimed to determine whether the new mutations also affect *HIF1A* or *TFAP2C* and finally lead to an abnormal expression of *VEGF* or *PITX2C* which play an important role in heart development.

## Materials and Methods

### Ethics statement

The study protocol conformed to the ethical guidelines of the 1975 Declaration of Helsinki and was approved by the Ethics Committee of the National Research Institute for Family Planning. Written informed consent was obtained from patients' parents or guardians.

### Subjects

The study population comprised 700 patients who were diagnosed with CHD based on anthropometric measurement, physical examination for malformation and dysmorphism, and radiological evaluation. The patients with a phenotype of VSD, TOF and ASD accounted for 43.71%, 8.42% and 12% respectively. 250 unrelated healthy children were used as controls. Peripheral blood was collected from each affected individual and their parents and controls were from 6 months to 12 years old and most of them volunteered to participate in the study.

We sequenced the whole *CITED2* ORF in 700 CHD patients (Table 1) and 250 healthy controls recruited from Lanzhou University, Beijing Children's Hospital, Zhengzhou Children's Hospital, Henan provincial Chest Hospital and Children's Hospital of Fudan University.

**Table 1:** Patients with congenital heart disease included in the study.

Phenotype	Total(n = 700)
Ventricular septal defect(VSD)	306
Tetralogy of Fallot(TOF)	59
Atrial septal defect(ASD)	84
Patent ductus arteriosus(PDA)	21
Pulmonal atresia or stenosis(PS)	21
double outlet right ventricle(DORV)	11
Aortic coarctation(COA)	4
Pulmonary hypertension(PH)	2
Other complex cardiac malformations	192

### Mutational analysis and bioinformatics

Genomic DNA was extracted from peripheral blood leukocytes using standard methods. The human *CITED2* gene is located on 6q24.1 and is encoded by two exons. One of the exons and splice sites of *CITED2* were amplified by polymerase chain reaction (PCR) using two pairs of *CITED2* gene-specific primers (Table 2). PCR products were sequenced using the appropriate PCR primers and the Big Dye Terminator Cycle Sequencing kit (Applied Biosystems, Foster City, CA, USA) and run on an automated sequencer, ABI 3730XL (Applied Biosystems), to perform mutational analysis.

**Table 2:** Primers used for PCR.

Name	Primer pair
Primers for *CITED2*	F CCGGCTGTGTTATGAGTGGTAG
	R AGTTGGGGGTTTGATTTCTTTC
Middle Primer for *CITED2*	TCGGAAGTGCTGGTTTGTC
Primers for P140S	F TGCCGGATTTGCACTCTGCTGCA GGCCAC
	R GTGGCCTGCAGCAGAGTGCAAAT CCGGCA
Primers for S183L	F GCTCTGGCAGCAGCTTGGGCGGCG
	R CGCCGCCCAAGCTGCTGCCAGAGC
Primers for S196G	F AACAGCGGCGGCGGCGGCGGCAGCG GCAACA
	R TGTTGCCGCTGCCGCCGCCGCCGCC GCTGTT
Primers for Ser161delAGCAGC	F TGCAACCCCAAGCACGGCGGCAGCA GCACC TGCAACCCCAAGCACGGCGGCAGCAGCACC
	R GGTGCTGCTGCCGCCGTGCTTGGGG TTGCA
Primers for Ser192_Gly193delAGCGGC	F CGCGGGCAGCAGCAACGGCGGCAGC GGCAGCGGCAACAT
	R ATGTTGCCGCTGCCGCTGCCGCCGTT GCTGCTGCCCGCG
pEGFP-*CIITED2*	F GGGGTACCATGGCAGACCATATGATG
	R CGGGATCCCGACAGCTCACTCTGCTGG
pCDNA3.1(+)-*CITED2*	F CGGGGTACCTATGGCAGACCATATGA TGGC
	R TGCTCTAGAGTCAACAGCTCACTCTGCTG
pCMX-GAL4-*CITED2*	F CGGATATCAATGGCAGACCATATGA TGGC
	R CTAGCTAGCTCAACAGCTCACTCTGCT
pCMX-GAL4-*HIF1A*	F CGGATATCAATGGAGGGCGCCGGCG
	R CTAGCTAGCTCAGTTAACTTGATCCAA AGCT
pCMX-VP16-*P300CH1*	F CGCGGATCCTATGGCCGAGAATGTGG TGGAAC
	R CTAGCTAGCCCAACGGGTGCTCCAGT CAAA
pCDNA3.1(+)-*HIF1A*	F CGGGGTACCTATGGAGGGCGCCGGC
	R TGCTCTAGATCAGTTAACTTGATCCAAAGC
pCDNA3.1(+)-*TFAP2C*	F CGGGGTACCACGCCGGACGCCATGTTG
	R TGCTCTAGACTCTCCTAACCTTTCTTC GTTCC
PGL3basic-*VEGF* promoter	F GGGGTACCTTTGGGTTTTGCCAGACT
	R CCGCTCGAGAGGAGGGAGCAGGAATAG
PGL3basic-*PITX2C* promoter	F GGGGTACCGGGGACAAAAGGACTTTC
	R CCGCTCGAGCCCTGTTGGCCTAACATC

### Site-directed mutagenesis and plasmid construction

Human *CITED2* and *HIF1A* cDNA were obtained from OriGene True-Clone, and *TFAP2C* cDNA was purchased from GeneCopoeia. *CITED2* mutations were constructed by using the Quick Change Lightning Site-Directed Mutagenesis kit (Strata gene, La Jolla, CA, USA). Then the introduced mutations were confirmed by DNA sequence.

The WT and mutant *CITED2* were amplified by PCR from cDNA and inserted into the pEGFP-N1 vector (BD Biosciences, Palo Alto, CA, USA). The ORF of *HIF1A* and *TFAP2C* were also amplified by PCR from cDNA and inserted respectively into the pcDNA3.1(+) vector (Invitrogen, Carlsbad, CA, USA) to create the expression plasmid pcDNA3.1-*HIF1A* and pcDNA3.1-*TFAP2C*.

A 1300-bp fragment of the p300-CH1, *PITX2C* promoter and an 870-bp segment of *VEGF* promoter amplified by PCR from Human genomic DNA were cloned respectively into the GAL4-pCMX vector and the luciferase reporter PLG3-basic vector. GAL4-*HIF1A* was constructed by cloning DNA fragments into GAL4-pCMX vector at the Ecorv and Nhel sites. All primers of the PCRS were list in Table 2.

The VP16-pCMX vector with the potent transactivating domain of HSV, the promoter pGL3-basic vector with 4×GAL4 DNA-binding sites and the GAL4-pCMX vector containing GAL4-DBD were provided by Dr. Ronald M. Evans (Salk Institute for Biological Studies, USA).

### Cell culture and transient transfection

293T and Hela cells were maintained in Iscove's modified Dulbecco's medium supplemented with 10% fetal bovine serum, 100 mg/ml penicillin, and 100 mg/ml streptomycin in a humidified atmosphere containing 5% CO_2_ at 37°C. Transfection was carried out using a standard calcium phosphate method or Lipofectamine 2000 (Invitrogen Corporation, Carlsbad, CA, USA).

### Subcellular localization

Hela cells were seeded in 12-well tissue culture plates 20 h prior to transfection at approximately 60% confluency. GFP-*CITED2* expression constructs containing wild-type and mutant *CITED2* were transfected using Lipofectamine 2000, according to the manufacturer's instructions. The empty vector pEGFP-N1 was transfected as a control. Forty hours after transfection, the cells were fixed and permeabilised in 4% paraformaldehyde for 15 min, 0.1% Triton X-100 for 20 min and the DNA was stained with 0.5 µg/ml DAPI for 3 min at room temperature. The cells were observed by fluorescence microscopy. All steps were operated in lucifugal conditions.

### Mammalian two-hybrid assay and transcriptional assays

Mammalian two-hybrid assay plasmids including pCMX-VP16-*p300*, TK promoter reporter plasmid, the Renilla luciferase control plasmid pREP7-RLu and pCMX- GAL4-*CITED2* (wild-type or mutant) or pCMX-GAL4-*HIF1A* were contransfected into 293T cells. Thirty hours after transfection, cells were washed and lysed in passive lysis buffer (Promega, Madison, WI, USA) and the transfection efficiency was normalised to paired Renilla luciferase activity by using the Dual Luciferase Reporter Assay System (Promega, Madison, WI, USA) according to the manufacturer's instructions.

In addition, the Dual LuciferaseReporter Assay System was used to study the effect of *CITED2* on the transcription of *VEGF* and *PITX2C*. Plasmids consisting of the Renilla luciferase control plasmid pREP7-RLu, pcDNA3.1-*CITED2* (wild-type or mutant), PGL3-*VEGF*-pro and pcDNA3.1-*HIF1A* or PGL3-*PITX2C*-pro and pcDNA3.1-*TFAP2C* were contransfected into 293T cells. Thirty hours after transfection, cells were treated the same way as above.

### Statistical analysis

The results represent the means of three independent experiments performed in triplicate, and the bars denote the S.D. The independent-samples t test was adopted to determine statistical significance of unpaired samples. All data were analyzed by Prism Demo 5 software.

## Results

### Genetic and bioinformatics analysis

From a total of 700 non-syndromic CHD patients, we identified five novel *CITED2* nucleotide alterations (two amino acid deletions and three amino acid substitutions, table3). Three mutations (c.C548T, c.A586G and c.574-59delAGCGGC) were found in one, one and four patients with Ventricular septal defect (VSD) respectively. One mutation (c.C418T) was detected in one patient with Tetralogy of Fallot (TOF) and another mutation (c.481–483delAGC) was detected in one patient with Artrial septal defect (ASD).

**Table 3:** Position of variations

Coding position	Amino acid position	Phenotype of mutation carrier
c.C418T	p. P140S,Pro-Ser	F4
c.C548T	p. S183L,Ser-Leu	VSD
c.A586G	p. S196G,Ser-Gly	VSD
c.481–483delAGC	p.Ser161delAGC	ASD
c.574–579delAGCGGC	p.Ser192_Gly193delAGCGGC	VSD

All potential pathogenic mutations have not been reported in the NCBI dbSNP and are not included in the 1000 Genome Project database (http://browser.1000genomes.org/).

**Figure 1:**
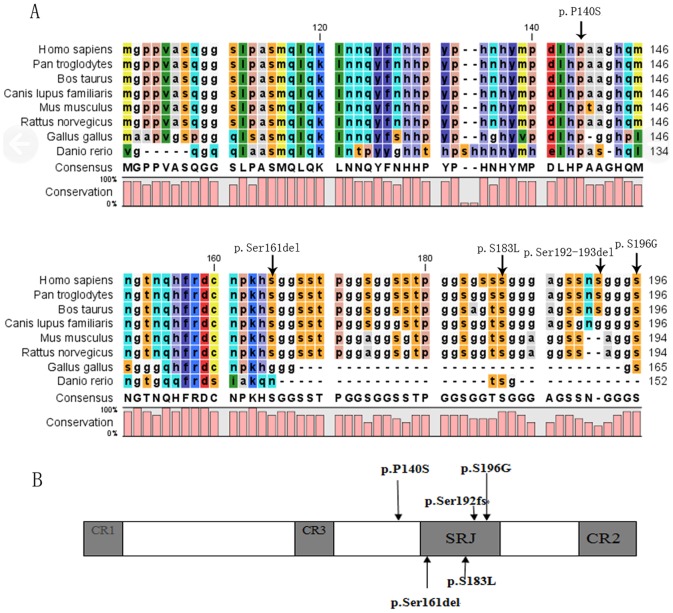
Structure of *CITED2*. **A**: Sequence alignment of *CITED2* proteins among several species. The figure showed that three acid substitutions were located at highly conserved regions among many species (human, chimpanzee, mice, dog, cattle, rat, chicken and zebrafish). **B**: Position of mutations in the *CITED2* protein identified in CHD patients. *CITED2* has three conserved regions CR1-3 and serine-glycine rich junction (SRJ). All other mutations were located in SRJ except p.P140S.

The result of sequence alignment of *CITED2* proteins among several species showed that three acid substitutions were located at highly conserved regions among different species (human, chimpanzee, mice, dog, cattle, rat, chicken and zebrafish) and two amino acid deletions were not located at highly conserved regions among these species (Figure 1).

### *CITED2* mutations decrease *HIF1A* repression leading to up-regulation of *VEGF* expression

**Figure 2:**
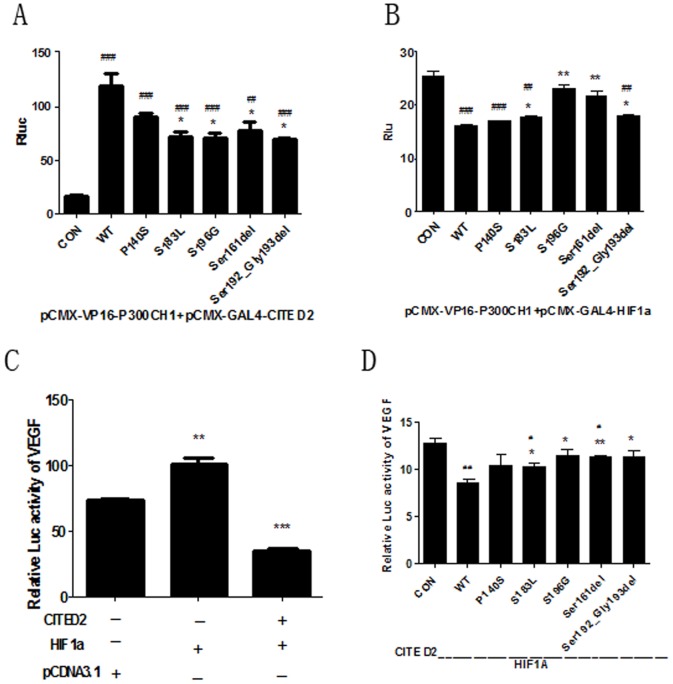
Effect of *CITED2* mutations on the transcriptional activation of *HIF1A* to its target gene *VEGF*. **A**: Effect of mutations on *CITED2*-*p300CH1* interactions. We cotransfected 293T cells with pCMX-VP16-*p300CH1*, TK promoter reporter plasmid, and the Renilla luciferase internal control plasmid, as well as empty vector pCMX-GAL4, GAL4-*CITED2* wild-type, and the mutants. The significance of differences was calculated using the independent-samples t test. (*p<0.05, **p<0.01 versus. wt-type, #p<0.05, ##p<0.01 versus.empty vector pCMX-GAL4.) **B**: Effect of mutations on *HIF1A*-*p300CH1* interactions. Cotransfection of pCMX-VP16-*p300CH1*, pCMX-GAL4-*HIF1A*, TK promoter reporter plasmid, and the Renilla luciferase internal control plasmid, as well as empty vector pcDNA3.1 (+), pcDNA3.1 (+)-*CITED2* wild-type, and the mutant. (* p<0.05, ** p<0.01 versus wt-type, # p<0.05, ## p<0.01 versus. empty vector pcDNA3.1 (+)) **C**: Effect of wt-type on the transcriptional activation of *VEGF*. Transfected the *VEGF* reporter plasmid and the expression vector for *HIF1A*, *CITED2* or pcDNA3.1 were transfected together in 293 T cells. The luciferase activity was normalized to Renilla activity.* p<0.05, **p<0.01 versus the untreated group (n = 3). **D**: Effect of *CITED2* mutants on transcription activation of *VEGF* compared with *CITED2*-wt. The rest report plasmids were same as above. (*p<0.05, **p<0.01 versus wt-type, #p<0.05, ##p<0.01 versus empty vector pcDNA3.1(+)). The results represent the means of 3 independent experiments performed in triplicate and the significance of differences was calculated using independent-samples t test.(*CITED2* =  *Cbp/p300*-interacting transactivator, with Glu/Asp-rich carboxy-terminal domain, 2, *HIF1A* =  Hypoxia Inducible Factor 1, *VEGF* =  vascular endothelial growth factor)

Two mammalian two-hybrid assays were used to evaluate whether the mutation affected the interaction between every two of *CITED2*, *p300CH1* and *HIF1A* (Figure 2). Cotransfection of both VP16- P300 and wild-type GAL4-*CITED2* with the TK promoter reporter plasmid led to a nearly 10-fold increase in luciferase activity compared with VP16-P300 and empty vector of CMX-GAL4 (t test, p<0.01). The luciferase activity of p. P140S mutant was even the same as the wt-type, However, cotransfection of VP16-P300 and the four mutants (p.S183L, p.S196G, p.Ser161delAGC, p.Ser192_Gly193delAGCGGC) GAL4-*CITED2* showed weakened luciferase activity (t test, p<0.05) (Figure 2A) compared with wt-type. These findings indicated that the four mutations diminished protein-protein interactions between p300 and *CITED2*, but the p.P140S mutant didn't alter the interactions.

Another mammalian two-hybrid assay was operated and analyzed to further evaluate whether the repression of *HIF1A* - p300 complex was influenced by *CITED2* mutation. The result showed that the luciferase activity of wt-type was only 60% of the control (t test, p<0.01) (Figure 2B). Compared with wild-type, the luciferase activity of mutants increased obviously except the p.P140S mutant. In conclusion, *CITED2* mutations weaken the *HIF1A* repression by diminishing the protein-protein interactions between *p300CH1* and *CITED2* on the one hand and by enhancing the interactions between *p300CH1* and *HIF1A* on the other hand.

As *HIF1A* can induce vascular endothelial growth factor (*VEGF*) potently, we supposed that *CITED2* mutations influenced the transcription of *VEGF* through their effect on *HIF1A*. This was confirmed by our dual luciferase assay (Figure 2C). Wild-type *CITED2* caused an approximately 32% decrease of activity compared with the control (t test, p<0.01). P140S showed no difference with wild-type in luciferase activity. As for the other four mutants, Ser161delAGCAGC showed an observable promotion of *VEGF*-promoter resulting in higher luciferase activity than wild-type (t test, p<0.01) and the rest mutants showed few differences compared with wt-type (t test, p<0.05) (Figure 2D).

### *CITED2* mutations impair *TFAP2C* coactivation resulting in abnormal transactivation of *PITX2C*

**Figure 3:**
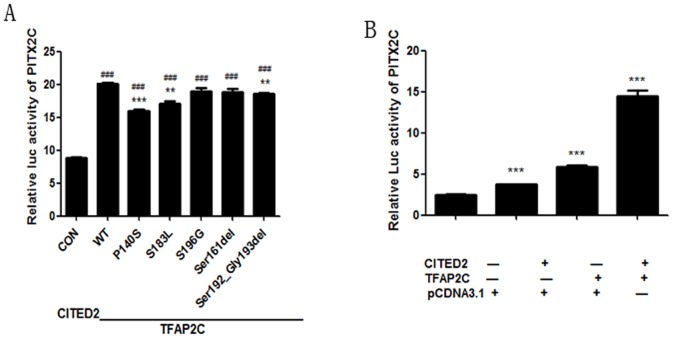
Effect of *CITED2* variants on the cooperation between *CITED2* and *TFAP2C* in the transactivation of the *PITX2C*. **A:** Effect of *CITED2* mutations on the transcription activation of *PITX2C*. (*p<0.05, **p<0.01 versus wt-type, #p<0.05, ##p<0.01 versus.empty vector pcDNA3.1(+)). **B:** *CITED2*-wt and *TFAP2C* working on the transcriptional activation of *PITX2C*. *PITX2C* reporter plasmid and the expression vector for *TFAP2C*, *CITED2*, or pcDNA3.1 alone were transfected respectively in 293 T cells. The luciferase activity was normalized to Renilla activity.(* p<0.05, **p<0.01 versus the untreated group (n = 3)).

As a transcriptional coactivator of *TFAP2*, *CITED2* influenced cardiac left-right patterning by regulating the left-right patterning Nodal-*PITX2C* pathway. *PITX2C* is a critical gene of the Nodal-*PITX2C* pathway and controls the location of heart and intestines in embryo. Our study showed that *CITED2* mutations resulted in decreased luciferase activity of PITX2 by diminishing the coactivation of *CITED2* and *TFAP2C*. The luciferase activity of three mutants were decreased obviously compared with wt. (p.P140S vs. wt-type 80% (t test, p<0.01), p.S183L vs. wt-type 85% (t test, p<0.01), p.Ser192_Gly193delAGCGGC vs. wt-type 92% (t test, p<0.01)) (Figure 3A). The rest two mutants coactivated *TFAP2C* to the same level as wt-type.

In addition, we designed another test to prove the *TFAP2* coactivation with *CITED2*. The result showed that cotransfection of empty vector of pcDNA3.1 (+) with the luciferase reporter PGL3-*PITX2C*-pro was the lowest in all groups including pcDNA3.1-*TFAP2C* or wt–type pcDNA3.1-*CITED2* only and both of them (Figure 3B).

In conclusion, *CITED2* mutations contributed to the abnormal transactivation of *PITX2C*.

### Impact of *CITED2* mutations on Subcellular Localization

To further study whether the functional changes are caused by changed subcellular localization of the protein, the transfections were performed using N-terminal GFP fusion constructs of wt and mutant *CITED2*, followed by fluorescence microscopy. The result indicated that the effects of *CITED2* mutations on *VEGF* and *PITX2C* were not caused by the incorrect localization of the protein. Whether in wt or mutant of *CITED2* the proteins were discovered mainly in nucleus and a lesser degree in the cytoplasm of Hela (Figure S1).

## Discussion

Previous researches of *cited2*-/-mice confirmed that *cited2* plays a critical role in the development of heart and is essential for the normal creation of the left–right axis. *Cited2*-/- embryos showed a series of cardiac malformations such as VSD, ASD, outflow tract abnormalities and abnormal heart looping.

We screened the coding region and splice sites of the *CITED2* gene in 700 Chinese CHD patients. Two potential pathogenic amino acid deletions (p.Ser161delAGCAGC and p.Ser192_Gly193delAGCGGC) and three potential pathogenic amino acid substitutions variants (p. P140S, p. S183L and p. S196G) were identified. These three regional highly conserved substitutions (conserved among Humans, chimpanzee, mice, dog, cattle, rat, chicken and zebrafish) were not identified in control group or the variant databases. Therefore, we supposed that these three mutations were possibly causative. Since, SRJ region is a research hot spot at present, the two potential pathogenic amino acid deletions in our study were found in SRJ region. As a result, the necessity of this study is highlight. Although the CHD phenotype was not seen in SRJ-deficient mice as observed in mutation carrying patients, we supposed that this could be due to species differences [23], [24]in the function of *CITED2*, or some other unidentified factors[25] might interact with *CITED2* and modify its phenotype. Alternatively, it is also possible that CHD were present earlier in life but spontaneously closed at a later time in SRJ-deficient mice.

Mammalian two-hybrid analysis permits the semi-quantitative assessment of protein-protein interactions occurring within living cells. Cotransfection of wt or mutant *CITED2* and *p300CH1* in 293Tcells, the binding between *CITED2* and *p300CH1* activated the TK report gene expression in vivo. The functional study greatly supported the hypothesis that the mutations are causative and might affect the formation of heart. The last four mutated proteins (p. S183L, p. S196G, p.Ser161delAGCAGC and p.Ser192_Gly193delAGCGGC) showed significantly decreased reporter gene activation ability compared with wt-type. However, an opposite phenomenon occurred by transfecting *p300CH1*, *HIF1A* and wt or mutant *CITED2* together in cells. Taken together,the results indicated that the four mutated proteins decreased the interaction between *CITED2* and *p300CH1* compared with wt-type,causing a weakened competitive binding to p300 CH1 of *CITED2*. The increased interaction between *HIF1A* and *p300CH1* could up- regulate the promoter activity of *VEGF* according to our dual luciferase experiment.

Our study also showed that three mutations decreased the consociation of *TFAP2C* and *CITED2* in the transactivation of *pitx2c*, an essential gene of the left–right axis establishment confirmed in mice and chick embryo. The mice experiments already indicated that knocking out *pitx2c* gene can lead to valve defects, body wall dysraphism, gastroschisis, ectopia cordis and other multiple organs polymorphous defects. In addition, there was no evidence that *CITED2* mutations were involved in the incorrect location of the protein in the subcellular localization experiment.

In conclusion, we identified five novel mutations among 700 CHD patients by screening the coding region and splice sites of the *CITED2* gene. To confirm our hypothesis that the mutations were pathogenic, we investigated the function and mechanism of them. Our study revealed that four mutations influenced the transcription regulatory properties of *VEGF* and three mutations reduced costimulation capacity to promote *PITX2C*. Further research showed that four *CITED2* mutations recovered the promoter activity of *VEGF* [26]caused by its decreased competitiveness with *HIF1A* to bind the *p300CH1*. Furthermore, three mutations also decreased the consociation of *TFAP2C* and *CITED2* in the transactivation of *PITX2C*. Our study confirmed that *CITED2* is a disease-causing gene of CHD and its mutations can result in the cardiac malformations.

## Supporting Information

Figure S1 Subcellular localization of *CITED2*. Localization of wild-type and mutant *CITED2* GFP-fusion protein in transfected Hela cells were observed by fluorescent microscope. The empty vector pEGFP-N1 was transfected as a control. All figures were drawn by fluorescence microscopy and Adobe Photoshop CS5.
(TIF)
