# Supplementary figures and images for: Variations of CITED2 Are Associated with Congenital Heart Disease (CHD) in Chinese Population
Source: PLoS One. 2014 May 21;9(5):e98157. doi: 10.1371/journal.pone.0098157 (PMC4029912; doi:10.1371/journal.pone.0098157)

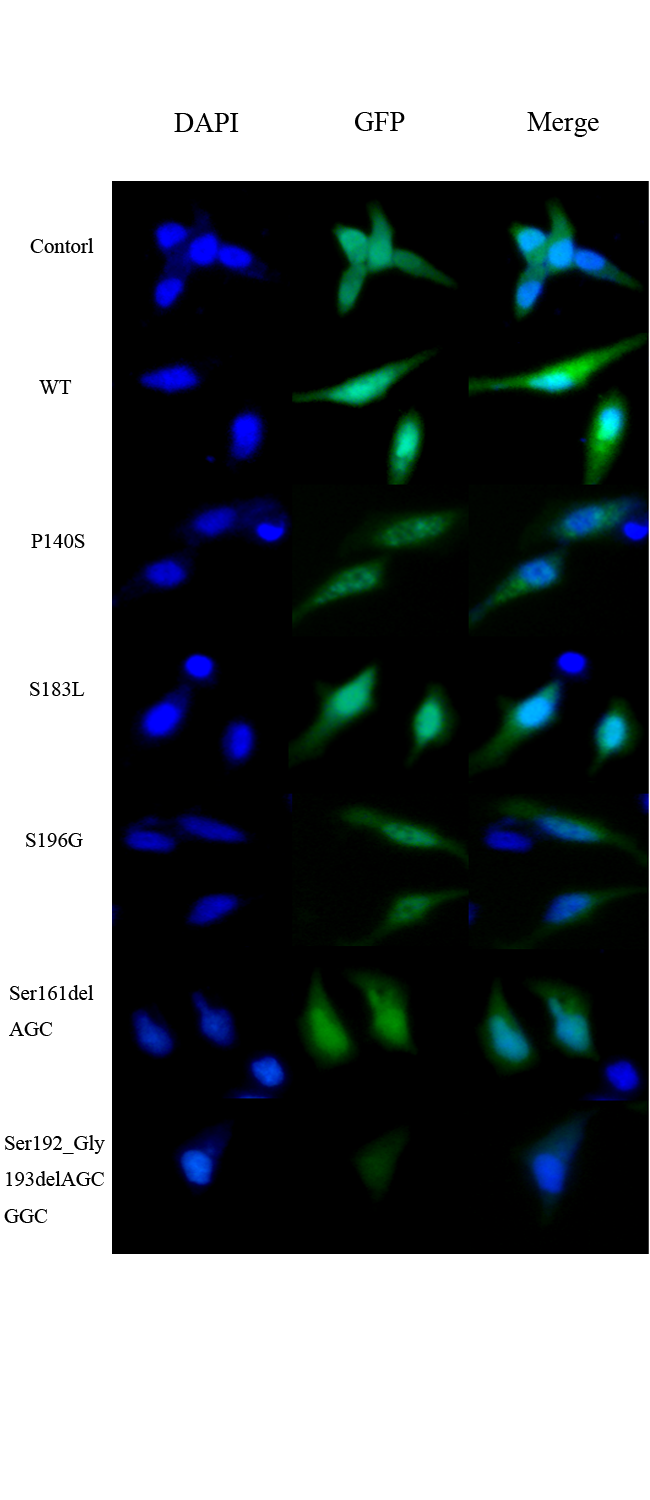

Supplement: Figure S1 — Subcellular localization of CITED2. Localization of wild-type and mutant CITED2 GFP-fusion protein in transfected Hela cells were observed by fluorescent microscope. The empty vector pEGFP-N1 was transfected as a control. All figures were drawn by fluorescence microscopy and Adobe Photoshop CS5. (TIF) [file pone.0098157.s001.tif]
